# Proteogenomic analysis demonstrates increased *bla*_OXA-48_ copy numbers and OmpK36 loss as contributors to carbapenem resistance in *Klebsiella pneumoniae*

**DOI:** 10.1128/aac.00107-25

**Published:** 2025-06-13

**Authors:** Lisa M. Meekes, Astrid P. Heikema, Manuela Tompa, Ana L. Astorga Alsina, Saskia D. Hiltemann, Andrew P. Stubbs, Lennard J. M. Dekker, Dimard E. Foudraine, Nikolaos Strepis, Johann D. D. Pitout, Gisele Peirano, C. H. W. Klaassen, W. H. F. Goessens

**Affiliations:** 1 Department of Medical Microbiology & Infectious Diseases, Erasmus MC, University Medical Center Rotterdam 6993, Rotterdam, the Netherlands; 2 Central Laboratory, Regional Institute of Gastroenterology and Hepatology “Prof. Dr. Octavian Fodor”, Cluj-Napoca, Romania; 3 3rd Medical Clinic, University of Medicine and Pharmacy, Cluj-Napoca, Romania; 4 3rd Surgical Clinic, University of Medicine and Pharmacy, Cluj-Napoca, Romania; 5 Department of Pathology and Clinical Bioinformatics, Erasmus MC, University Medical Center Rotterdam 6993, Rotterdam, the Netherlands; 6 Department of Neurology, Clinical and Cancer Proteomics, Erasmus MC, University Medical Center Rotterdam 6993, Rotterdam, the Netherlands; 7 Department of Pathology and Laboratory Medicine, Cumming School of Medicine, University of Calgary 70401, Calgary, Alberta, Canada; 8 Department of Microbiology and Infectious Diseases, Cumming School of Medicine, University of Calgary, Calgary, Alberta, Canada; University of Fribourg, Fribourg, Switzerland

**Keywords:** antimicrobial resistance, mass spectrometry, porins, OXA-48, *Klebsiella*, OmpK36

## Abstract

Antimicrobial resistance arises from complex genetic and regulatory changes, including single mutations, gene acquisitions, or cumulative effects. Advancements in genomics and proteomics facilitate a more comprehensive understanding of the mechanisms behind antimicrobial resistance. In this study, 74 clinically obtained *Klebsiella pneumoniae* isolates with increased meropenem and/or imipenem MICs were characterized by broth microdilution and PCR to check for the presence of carbapenemase genes. Subsequently, a representative subset of 15 isolates was selected for whole-genome sequencing (WGS) by Illumina and Nanopore sequencing, and proteomic analysis by liquid chromatography-tandem mass spectrometry (LC-MS/MS) to investigate the mechanisms underlying the differences in carbapenem susceptibility of *Klebsiella pneumoniae* isolates. Identical techniques were applied to characterize four mutants obtained after sequential meropenem exposure. We demonstrated that in clinically obtained isolates, increased copy numbers of *bla*_OXA-48_-containing plasmids, combined with OmpK36 loss, contributed to high carbapenem MICs without the involvement of OmpK35 or other porins or efflux systems. In the meropenem-exposed mutants, increased copy numbers of *bla*_CTX-M-15_ or *bla*_OXA-48_-containing plasmids, combined with OmpK36 loss, were demonstrated. The OmpK36 loss resulted from the insertion of IS1 transposable elements or partial deletion of the *ompK36* gene. Additionally, we identified two mutations, C59A and C58A, in the DNA coding the copA antisense RNA of IncFII plasmids and multiple mutations of an IncR plasmid, associated with increased plasmid copy numbers. This study demonstrates that by combining WGS and LC-MS/MS, the effect of genomic changes on protein expression related to antibiotic resistance and the mechanisms behind antibiotic resistance can be elucidated.

## INTRODUCTION

Antimicrobial drug resistance is one of the major threats to human health (1). Especially bacteria resistant to carbapenem antibiotics are a threat, as carbapenem antibiotics are among the last-resort treatments (2, 3). In general, bacteria become resistant to carbapenem antibiotics by acquiring a carbapenemase gene, of which *Klebsiella pneumoniae* carbapenemase, New Delhi metallo-β-lactamase (NDM), Verona integron-encoded metallo-β-lactamase, imipenemase, and carbapenemase-type OXA-48-like β-lactamases are most prevalent in Enterobacterales (2). In contrast to the other mentioned carbapenemase genes, OXA-48 and OXA-48-like enzymes display only weak carbapenemase activity (4–6). As a result, Enterobacterales harboring these *bla*_OXA-48_ enzymes generally demonstrate low carbapenem MICs (4, 5); however, they are able to display MICs above the resistance (R) breakpoint. It has been shown that in *bla*_OXA-48_-positive Enterobacterales, high carbapenem MICs are linked to the combination of *bla*_OXA-48_ and porin loss (4, 7).

In addition to the acquisition of carbapenemase genes, Enterobacterales can become resistant to carbapenems by other mechanisms. In both *Klebsiella pneumoniae* and other Enterobacterales, a combination of porin loss and increased production of extended-spectrum β-lactamases (ESBLs) or AmpC has been reported as a cause of carbapenem resistance (7–10). The increased production of ESBL or AmpC is a prerequisite for meropenem or imipenem resistance, as porin loss alone results only in marginally elevated meropenem and/or imipenem MICs still within the susceptible range (7, 11, 12). These noncarbapenemase-producing carbapenem-resistant *Enterobacterales* (non-CP-CRE) have been reported to be selected *in vivo* during carbapenem therapy (8, 10). In recent surveillance programs, 19%–52% of clinical carbapenem non-susceptible *Enterobacterales* were in fact non-CP-CRE (13, 14).

Previous studies on carbapenem resistance have relied on various techniques such as quantitative PCR, hydrolysis assays, knockouts, and SDS-PAGE (7, 9–12, 15, 16) to demonstrate β-lactamase expression and porin loss. However, with recent advancements in genomics and proteomics, a more comprehensive approach to understanding the role of these mechanisms is now possible. Therefore, we first aimed to elucidate the mechanisms underlying the differences in carbapenem MICs in a subset of clinically obtained Romanian *K. pneumoniae* isolates by combining genomic and proteomic analysis. Second, we simulated these findings *in vitro* by exposing a selected number of the clinical isolates to several rounds of meropenem exposure. Proteogenomic analysis of the mutants in comparison to the native clinical isolates not only demonstrated the combination of increased plasmid copy numbers and porin loss but also enabled us to study the genetic mechanisms underlying these changes and their effects on protein expression of both porins and β-lactamases.

## RESULTS AND DISCUSSION

### Isolate characterization

Seventy-four clinically obtained *Klebsiella pneumoniae* isolates with increased MICs to meropenem and/or imipenem were selected at the Regional Institute of Gastroenterology and Hepatology “Prof. Dr. Octavian Fodor” in the Cluj-Napoca region of Romania in 2013. PCR revealed that 59 of the isolates (80%) harbored a prevalent carbapenemase gene, 57 isolates (77%) harbored *bla*_OXA-48-like_, and 2 isolates (3%) harbored both *bla*_OXA-48-like_ and *bla*_NDM_ (Table S1). Both the *bla*_OXA-48-like_ negative and *bla*_OXA-48-like_ positive isolates displayed a wide range in carbapenem MICs (Table S1). Due to this wide range in carbapenem MICs and the high prevalence of *bla*_OXA-48_-positive isolates, this previously unpublished collection of consecutively collected isolates was well suited to study the mechanisms causing differences in carbapenem MICs. Therefore, a representative subset of 15 isolates was selected based on carbapenem MIC and MLVA type to be analyzed further by whole-genome sequencing and liquid chromatography-tandem mass spectrometry (LC-MS/MS). At a later stage, four of these isolates were selected for resistance selection during several rounds of meropenem exposure.

### Genetic characterization of clinically obtained isolates

All 15 selected isolates contained one copy of *bla*_SHV-1_, *bla*_SHV-11_, or *bla*_SHV-28_ on the chromosome. In total, 14 isolates were *bla*_CTX-M-15_ positive; five isolates harbored *bla*_CTX-M-15_-positive plasmids, six isolates harbored two to three chromosomal *bla*_CTX-M-15_ copies, and three isolates harbored both *bla*_CTX-M-15_-positive plasmids and one to three chromosomal *bla*_CTX-M-15_ copies. The 10 *bla*_OXA-48-like_ positive isolates harbored a *bla*_OXA-48_ gene on an IncL(pOXA-48) plasmid (Fig. S1; Table S2).

### Increased plasmid copy numbers and ompK36 expression are associated with high carbapenem MICs

**Fig 1:**
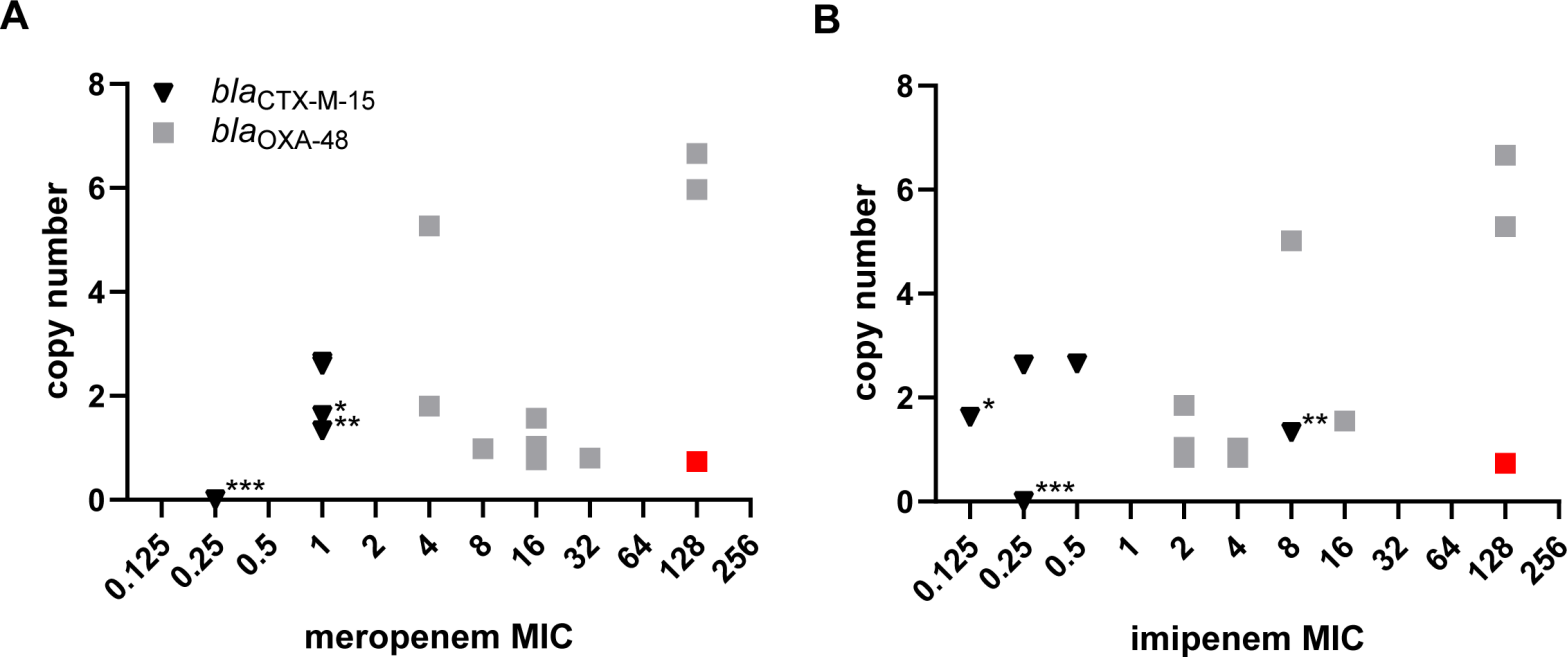
Copy number of the plasmids carrying *bla*_OXA-48_ or *bla*_CTX-M-15_ versus (A) meropenem MIC and (B) imipenem MIC. For *bla*_OXA-48_-positive isolates, the plasmid copy number of *bla*_OXA-48_ is plotted. For *bla*_OXA-48_-negative isolates, the plasmid copy number of *bla*_CTX-M-15_ is plotted. The number of *bla*_CTX-M-15_ copies on the chromosome of the *bla*_OXA-48_ negative isolates is indicated by the number of asterisks (*). The isolate harboring both *bla*_OXA-48_ and *bla*_NDM_ is depicted in red.

**Fig 2:**
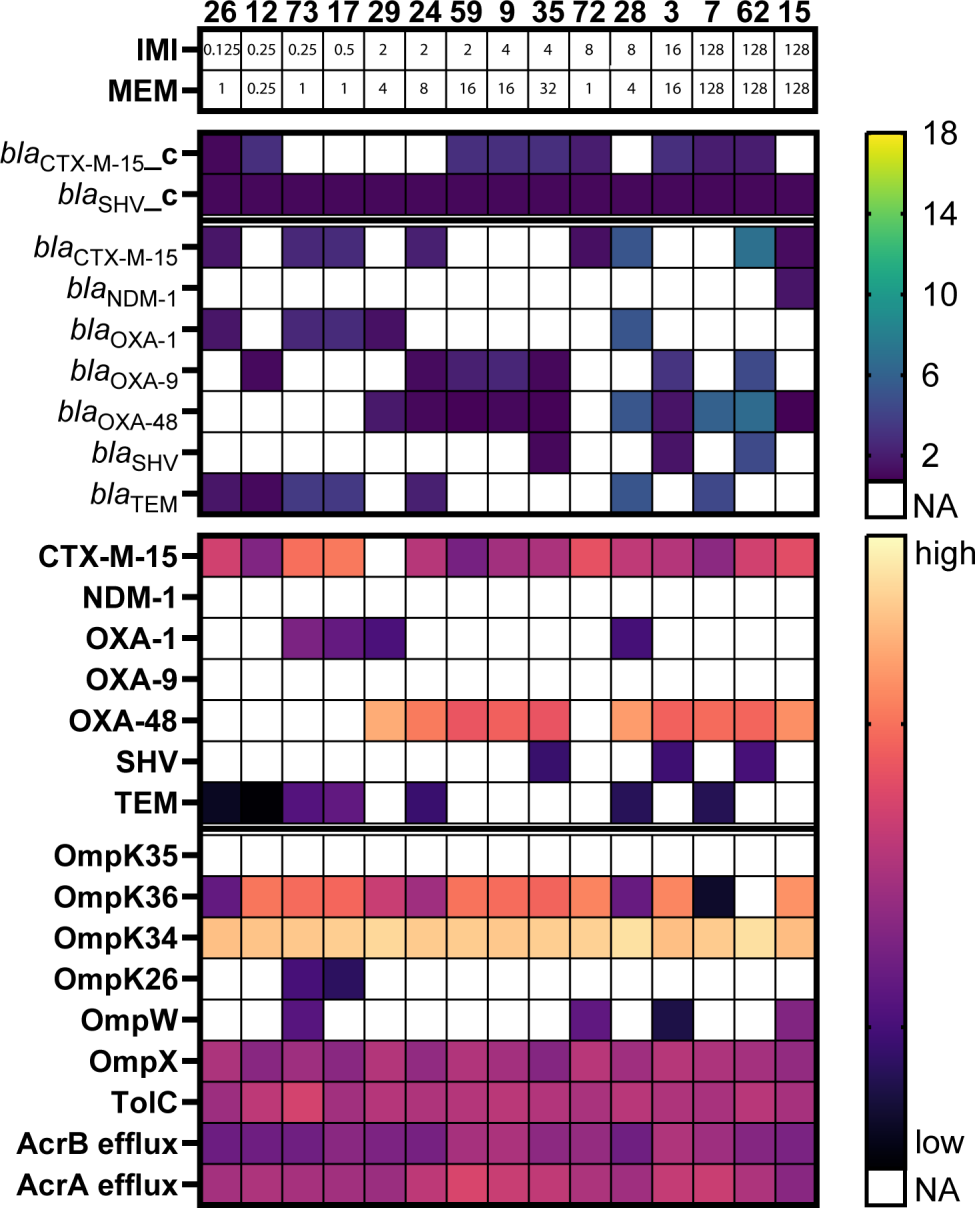
Plasmid copy number of β-lactamase genes and protein expression of β-lactamases, porins, and efflux systems for the 15 clinically obtained isolates. For each isolate, absent genes and proteins are depicted in white. Plasmid copy number of present β-lactamase genes and 2 log-transformed protein quantification are shown as a heatmap. The presence of a β-lactamase gene on the chromosome is depicted with the suffix “_c.” The different SHV and TEM β-lactamase enzymes are displayed as SHV and TEM-β-lactamase instead of specific gene variant, e.g., SHV-1. Isolates are clustered by carbapenem MICs. IMI, imipenem MIC; MEM, meropenem MIC.

The 15 clinical isolates were purposefully selected to reflect the variety in the entire collection. Remarkably, isolates 3, 24, 35, 59, and 62 contained a 100% identical IncL(pOXA-48) plasmid, despite imipenem MICs ranging from 2 to 128 µg/mL and meropenem MICs from 8 to 128 µg/mL (Fig. S1; Table S2). Both for these five isolates and the other *bla*_OXA-48_-positive isolates, a trend was observed between a high plasmid copy number and high carbapenem MICs (Fig. 1; Table S2). However, plasmid copy number did not explain all MIC variation. For example, both isolates 28 and 29 had meropenem MICs of 4 µg/mL, despite *bla*_OXA-48_ copy numbers of 5.27 and 1.8, respectively. In contrast, isolate 7, with a similar *bla*_OXA-48_ copy number (5.97) as isolate 28, showed a higher meropenem MIC of 128 µg/mL, likely due to its low OmpK36 expression compared to isolates 28 and 29, which had a 5.1- and 28.8-fold higher OmpK36 expression, respectively (Fig. 2; Table S2). The high carbapenem MICs of isolate 15, despite low IncL(pOXA-48) copy number and high OmpK36 expression, can be attributed to the presence of an additional NDM β-lactamase known for their efficient hydrolysis of carbapenem antibiotics (17) (Fig. 2). For the *bla*_OXA-48_-negative isolates, no trends were detected between carbapenem MICs and plasmid copy number, or porin and efflux pump expression (Fig. 1; Table S2). Overall, low OmpK36 expression and high copy numbers of *bla*_OXA-48_-containing plasmids seem to contribute to high imipenem and meropenem MICs.

### Meropenem resistance selection

**Fig 3:**
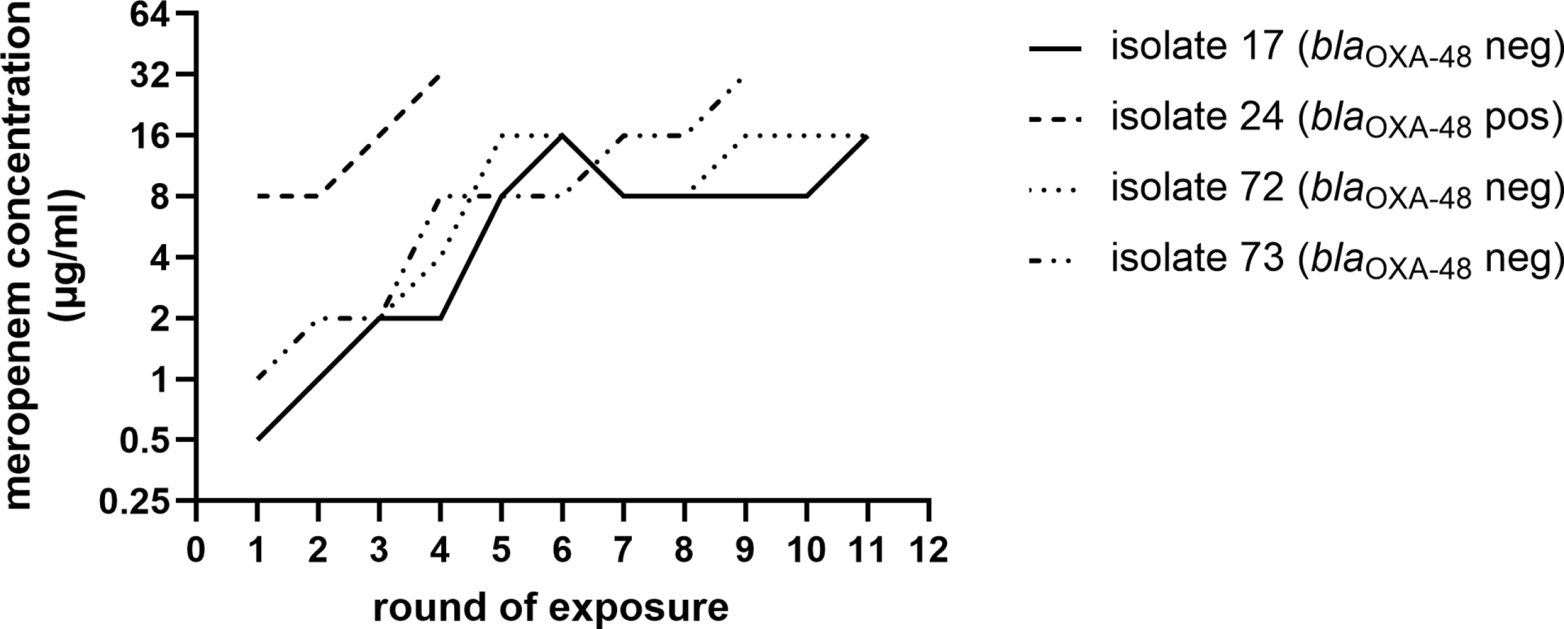
Meropenem resistance selection of four *K. pneumoniae* isolates. *bla*_OXA-48_-positive and *bla*_OXA-48_-negative isolates were exposed to increasing concentrations of meropenem until growth was obtained within a meropenem concentration of 32 µg/mL or the isolate was exposed for a total of 11 days. The highest meropenem concentration (µg/mL) at which the generation of each isolate could grow is plotted against the round of meropenem exposure.

To further study the genetic mechanisms underlying the low porin expression and increased plasmid copy number, four isolates with relatively low meropenem MICs were exposed to sequential rounds of increasing meropenem concentrations. We included three *bla*_OXA-48_-negative isolates to study whether porin loss and increased plasmid copy numbers could also explain the variability of meropenem MICs for *bla*_OXA-48_-negative isolates. Exposing the *bla*_OXA-48_-positive isolate to consecutive rounds of meropenem resulted in an isolate able to grow at a meropenem concentration of 32 µg/mL within four generations (Fig. 3). One *bla*_OXA-48_-negative isolate was able to grow at a meropenem concentration of 32 µg/mL after 9 days of meropenem exposure despite the absence of a carbapenemase; the other two grew at 16 µg/mL after 11 rounds of meropenem exposure (Fig. 3). Subsequently, one colony of the final generation of each meropenem-exposed isolate was selected for broth microdilution (BMD) MIC determinations, as well as for WGS and LC-MS/MS analysis.

### Genetic mutations associated with increased plasmid copy number

**Fig 4:**
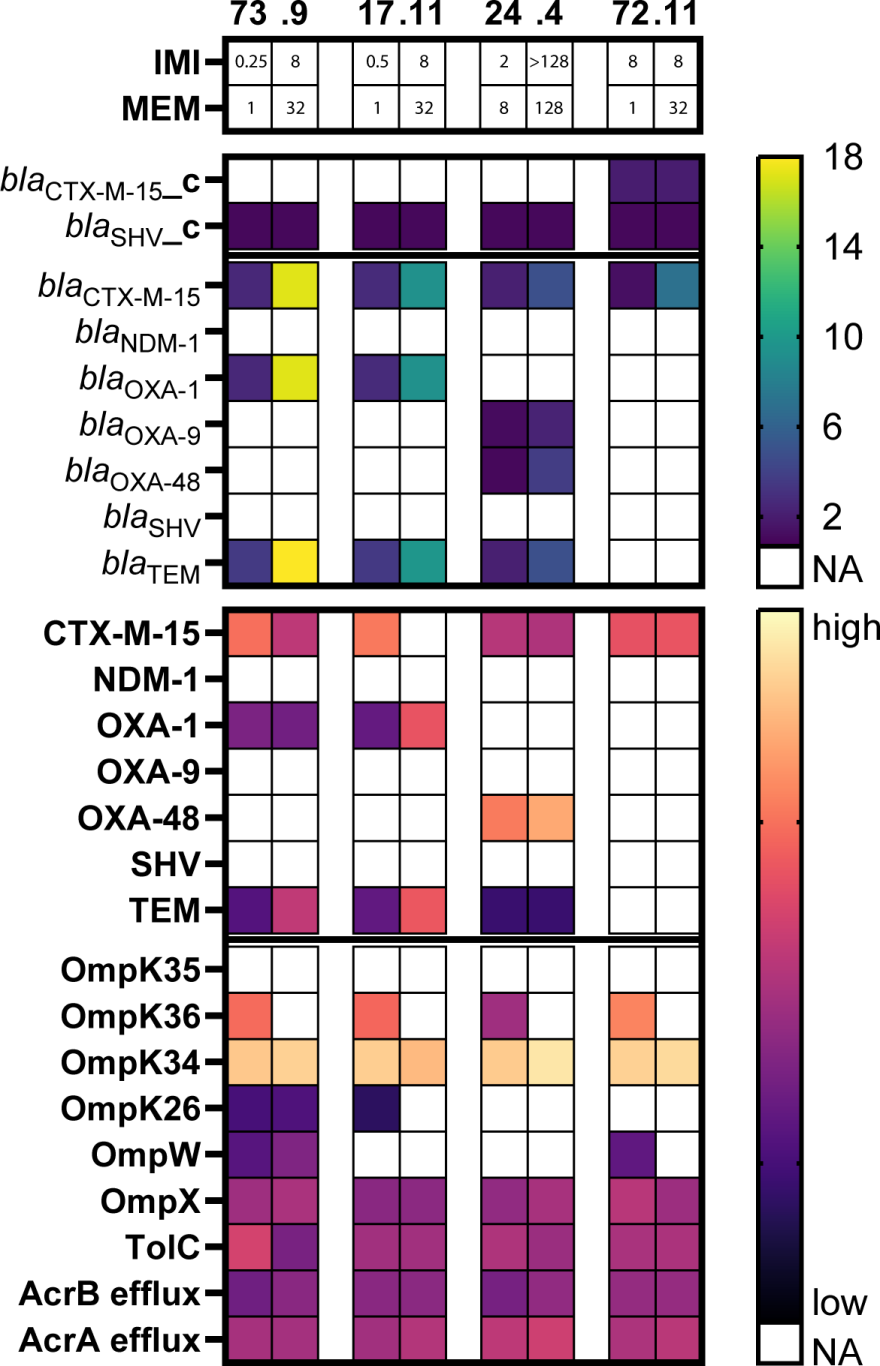
Plasmid copy number of β-lactamase genes and protein expression of β-lactamases, porins, and efflux systems for the four native isolates (left) and their corresponding meropenem-exposed isolates (right). For each isolate, absent genes and proteins are depicted in white. Plasmid copy number of present β-lactamase genes and 2 log-transformed protein quantification are shown as a heatmap. The presence of a β-lactamase gene on the chromosome is depicted with the suffix “_c.” The different SHV and TEM β-lactamase enzymes are displayed as SHV and TEM-β-lactamase instead of specific gene variant, e.g., SHV-1. Isolates are clustered by carbapenem MICs. IMI, imipenem MIC; MEM, meropenem MIC.

We assessed changes in both plasmid copy number and protein expression levels of the meropenem-exposed isolates compared to the corresponding native, clinically obtained isolates. For both the *bla*_OXA-48_-positive and *bla*_OXA-48_-negative isolates, our data showed increased plasmid copy numbers as the cause of increased β-lactamase production (Table S3), consistent with previous reports in *Escherichia coli* (18), rather than the recently described gene amplification (13). The *bla*_CTX-M-15_ containing IncFII plasmids of the two *bla*_OXA-48_-negative meropenem-exposed isolates 17.11 and 73.9 increased 6.7- and 3.4-fold after meropenem exposure (Fig. 4; Table S3). The hybrid assemblies of the IncFII plasmids showed a single base pair substitution in the coding sequence of antisense RNA copA, C59A in isolate 17.11 and C58A in isolate 73.9 (Fig. S2). CopA antisense RNA forms a complex with the complementary copT antisense RNA, which prevents plasmid replication (19). The single-nucleotide polymorphisms (SNPs) found in our meropenem-exposed isolates are located in the copA RNA loop that binds copT (19, 20). SNPs at the same nucleotide position have been associated with a three- to fourfold increase in plasmid copy number in the study of Brady et al. (20). Our experiments show that these SNPs also occur in clinically obtained *K. pneumoniae* isolates during meropenem exposure.

The *bla*_CTX-M-15_-containing IncR plasmid of isolate 72.11 increased 5.3-fold after sequential meropenem exposure (Fig. 4; Table S3). In the IncR plasmid of isolate 72.11, three mutations occurred: a substitution was detected in retron-type RNA direct DNA polymerase; a small 90 amino acid hypothetical protein upstream of the IncR replicon was deleted; and in DNA polymerase I, a 481 nucleotide long element was inserted, leading to gene disruption (Fig. S2). The inserted element consisted of 13 copies of a highly similar 37 bp long sequence, which was inserted in a part of the gene already comprising 17 copies of this sequence. Both the 17 copy-long sequence of the initial clinical isolate 72 and the 30 copy-long sequence of the meropenem-exposed isolate 72.11 were already found in GenBank with 100% similarity, indicating the involvement of a yet unknown mechanism rather than repeat duplication and spontaneous single-nucleotide mutations. As the insertion resulted in a frameshift, its role in the increased plasmid copy number remains unclear. The deletion of the hypothetical protein upstream of the origin of replication might also influence plasmid replication.

For the IncL(pOXA-48) plasmid of the meropenem-exposed isolate 24 and mutant 24.4, no genomic mutation was identified despite an increase in plasmid copy number from 0.98 to 3.74 (Fig. 4; Table S3). In addition, the IncL(pOXA-48) plasmids of five of the clinically obtained isolates were 100% identical to each other, despite the plasmid copy number ranging from 0.77 to 6.66. The variation in plasmid copy number despite 100% identical plasmids and similar growth conditions prior to proteomic analysis suggests a role for bacterial host factors or factors on other plasmids regulating plasmid copy number.

### OmpK36 loss is crucial for high carbapenem MICs

Protein expression analysis by LC-MS/MS identified 360 proteins with altered expression levels, exhibiting more than a fourfold increase or decrease in abundance in at least one of the meropenem-exposed isolates compared to its corresponding native isolate. However, only the expression of universal stress protein G was increased in all four isolates, and only the expression of OmpK36 and the osmotically inducible peroxide reductase OsmC family protein involved in oxidative stress was decreased in all four isolates. OmpK36 was undetectable by LC-MS/MS for all four meropenem-exposed isolates compared to high expression of OmpK36 by the four native clinically obtained isolates (Fig. 4).

Although OmpK36 loss due to transposon insertions is hypothesized by Ma et al. (21) to be most favorable for bacteria due to the reversible nature of transposon insertion, we found a range of mutations leading to OmpK36 loss, as previously reported (7, 16, 22, 23). In the *ompK36* region of the four meropenem-exposed isolates, different mutations occurred. In the *ompK36* gene of isolate 17.11, a 318 bp deletion was observed, and in isolate 72.11, a large 1,676 bp DNA fragment was deleted that comprised the N-terminus of *ompK36*, the upstream gene encoding a hypothetical protein, and a part of the upstream gene encoding a phosphotransferase. In the *ompK36* gene of isolates 24.4 and 73.9, an IS1 transposable element was inserted. Similarly, in isolate 62, two IS1 transposable elements were inserted downstream of the start of *ompK36*, indicating the role of these insertion elements in OmpK36 loss in clinical isolates.

### OmpK35 mutations in the majority of clinically obtained isolates

The other major *K. pneumoniae* porin OmpK35 was not detected in any of the clinically obtained or meropenem-exposed isolates. Mutations in *ompk35* were detected in 13 of the 15 clinically obtained isolates and 3 of the 4 meropenem-exposed mutants. A G947A substitution, leading to a premature stop codon, was detected in both isolate 17 and the meropenem-exposed isolates 17.11 and 73.9. The other 12 clinically obtained isolates and meropenem-exposed isolate 72.11 had small deletions ranging from 1 to 11 nucleotides, leading to frameshift errors and non-functional proteins, explaining the lack of OmpK35 detection by LC-MS/MS. A similar high frequency of *ompK35* mutations was recently reported by Strahilevitz et al. (24), who found *ompK35* alterations in 60 of 69 non-carbapenemase-producing *Klebsiella pneumoniae* ST 395 isolates. While OmpK36 loss comes with a considerable fitness cost, OmpK35 loss has a much lower fitness cost under highly nutritious *in vivo* conditions and is hypothesized to be a new adaptation to human colonization (25). For the other two clinically obtained isolates (24 and 73) and the meropenem-exposed isolate 24.4, no mutations were found in the *ompk35* gene or the promoter region. For these isolates, OmpK35 may have been undetectable due to culture conditions, as Tsai et al. (9) and Fajardo-Lubián et al. (25) showed that the expression of OmpK35 is reduced in MH II broth *in vitro*.

### No role for other porins of efflux systems in increased carbapenem MICs

Besides the major porins OmpK35 and OmpK36, *K. pneumoniae* isolates express multiple other porins and efflux pumps that might influence antibiotic concentrations in the bacterial cell. Compared to the strong evidence on the role of OmpK35 and OmpK36 in carbapenem resistance, the role of the other *K. pneumoniae* porins and efflux pumps remains less clear. The phosphate-regulated PhoE porin was previously found to be upregulated in *K. pneumoniae* isolates, which are both OmpK35 and OmpK36 deficient to compensate for the loss of nutrient influx in the bacterial cell (25). However, PhoE was not detected in any of our strains using LC-MS/MS. This may be the result of the continuous meropenem exposure of our strains, as Kaczmarek et al. (26) found that increased PhoE expression decreases carbapenem MICs. In addition, the recent observation by Nicolas-Chanoine et al. (7) that increased expression of both the AcrAB and the OqxAB efflux pump causes elevated imipenem MICs in *bla*_OXA-48_-positive isolates was not found for the *bla*_OXA-48_-positive isolates in the present study. Instead, we found no role for other porins or efflux pumps apart from OmpK36 as the cause of increased carbapenem MICs in accordance with the results of Doumith et al. (16), Hamzaoui et al. (22), and Saw et al. (15) (Fig. 2).

By combining genomic and proteomic analysis, our study provides valuable insights into the mechanisms of carbapenem resistance; however, a few limitations must be considered. First, as *bla*_OXA-48_ and *bla*_CTX-M-15_ were described as causes of carbapenem resistance in *K. pneumoniae* (4, 7, 8), we focused the analysis of the proteomic data on these β-lactamase genes. However, one study by Adler et al. (27) identified *bla*_TEM-1_ and, to a lesser extent, *bla*_OXA-1_ as contributors to increased meropenem MICs in an *E. coli* isolate. In five of our isolates, *bla*_CTX-M-15_ was present on the same plasmid as *bla*_TEM-1_ and/or *bla*_OXA-1_. For these isolates, we cannot exclude an additional or synergistic effect of the increased plasmid copy number and increased expression of *bla*_TEM-1_ and/or *bla*_OXA-1_ on the increased carbapenem MICs.

Second, protein quantification using LC-MS/MS in data-dependent acquisition mode is a semi-quantitative method. While the changes in β-lactamase and porin expression could already be detected with this semi-quantitative method, future studies combining genomics and proteomics could conduct the experiments using an MS method with absolute quantification or the use of stable isotope labeling for quantification to further characterize the alterations in protein expression.

### Conclusion

In conclusion, the differences in carbapenem MICs of *bla*_OXA-48_ or *bla*_CTX-M-15_-positive *K. pneumoniae* isolates can be explained by increased plasmid copy numbers of plasmids carrying these β-lactamase genes in combination with OmpK36 loss. Furthermore, we identified not only the various mutations causing the OmpK36 loss but also the specific SNPs in the coding sequence of the regulatory copA antisense RNA and the multiple mutations in the IncR plasmid, which might be involved in the increased plasmid copy numbers. Our study shows that combining hybrid genomics and proteomics to investigate antimicrobial resistance allows for the identification of the specific genomic changes driving antibiotic resistance and their association with changes in protein expression. It also emphasizes the importance of diagnostics on porin expression, as porin loss causes *K. pneumoniae* isolates with and without carbapenemase genes to become carbapenem resistant within a few days of meropenem exposure.

## MATERIALS AND METHODS

### Bacterial isolates

Seventy-four clinical *K. pneumoniae* isolates with an MIC ≥ 1 µg/mL for imipenem and/or an MIC ≥ 0.25 µg/mL for meropenem were collected during an outbreak at the Regional Institute of Gastroenterology and Hepatology “Prof. Dr. Octavian Fodor” in the Cluj-Napoca region of Romania in 2013. Isolates were routinely grown overnight at 37°C on Tryptic Soy Agar 5% sheep blood agar plates (TSA-BA, Becton Dickinson, Heidelberg, Germany). Confirmation of bacterial species was performed using MALDI-TOF (MALDI Biotyper, Bruker, Bremen, Germany) following the manufacturer’s instructions.

#### MIC determination by broth microdilution

Antibiotic susceptibility was determined by BMD method and performed according to the guidelines of the European Committee on Antimicrobial Susceptibility Testing (28, 29) using imipenem, meropenem (Sigma-Aldrich, Munich, Germany), and round-bottom 96-well plates (Corning, NY, USA). The reference strain *E. coli* ATCC 25922 was included during each MIC testing. MIC values were interpreted according to the EUCAST clinical breakpoints (30).

#### Selection of isolates

Real-time PCR assays were performed using established procedures to detect the carbapenemases *bla*_OXA-48-like_, *bla*_KPC_, *bla*_NDM_, and *bla*_VIM_ (31). Subsequently, MLVA analysis was performed as previously described (32). Out of the 74 isolates, a representative subset of 15 isolates was selected for additional genomic and proteomic analyses based on MIC value, MLVA type, and presence or absence of carbapenemase genes.

### Genomics

The selected 15 isolates were sequenced using both Illumina and Nanopore technology. For Illumina sequencing, DNA was isolated using MagNA Pure 96 DNA and viral NA small volume kits (Roche, Basel, Switzerland) on a MagNA Pure 96 (Roche). DNA extracts were treated with RNase prior to sequencing using Illumina technology, generating 150-nt paired-end reads (Novogene, Hong Kong, China). For Nanopore sequencing, DNA was extracted with Qiagen Genomic tips 20/G in combination with the Genomic DNA Buffer Set (Qiagen, Hilden, Germany). Libraries were constructed using the Rapid Barcoding kit (Oxford Nanopore Technologies [ONT], Oxford, UK) and sequenced on a GridION using an R-9.4 flowcell (ONT).

From the Nanopore and Illumina sequence reads, hybrid genome assemblies were generated with an in-house developed Galaxy pipeline (33) containing established bioinformatics tools such as Unicycler (34) (assembly), Prokka (35) (annotation), staramr (36) (scans genomes against ResFinder, CARD, PointFinder, and PlasmidFinder databases), and JBrowse (37) to browse through the assembled and annotated genomes (Galaxy version 0.4.8.0) and further analyzed with Geneious version 2022.2.2 software (38). Species identification of all sequenced isolates was confirmed by running TYGS analysis (39). After confirming full and uniform coverage of the plasmid sequences in JBrowse, the copy numbers of the plasmids, relative to the chromosome, were taken from the Unicycler assembly files. To validate the plasmid copy number estimates from the Unicycler assembly, we mapped sequence reads to the target gene, the contig containing the gene, and the entire genome. Copy numbers were calculated relative to the whole assembly, and the results showed good agreement (± ~25%) with those from the Unicycler assembly. The Mauve alignment tool (40) in Geneious Prime was used to analyze the plasmids for alterations such as insertions, deletions, and SNPs. Conventional MLST types were determined based on the Pasteur scheme (41).

### Proteomics

After overnight incubation on TSA-BA, one inoculation loop of each bacterial isolate was inoculated into 5 mL of MH II broth (MHB) and incubated overnight at 37°C at 150 rpm. The next day, 1 mL of the broth culture was centrifuged for 5 min at 21,000 *g* at 4°C and washed with 900 µL phosphate-buffered saline. Sample preparation, LC-MS/MS measurements, and analysis were performed using previously described methods (42). In short, the bacterial pellets were lysed with lysis buffer (50 mM triethylammonium bicarbonate buffer, 7.5 mM dithiothreitol, 5% acetonitrile, and 5% [wt/wt] sodium deoxycholate), incubated at 80°C, and sonicated. Afterward, each lysate was consecutively reduced using 50 mM dithiothreitol and alkylated using 100 mM iodoacetamide. Subsequently, the proteins were precipitated by washing twice with cold acetone. The obtained pellets were dissolved and sonicated in digest buffer (50 mM triethylammonium bicarbonate buffer, 5% acetonitrile, and 0.5% [wt/wt] sodium deoxycholate). After overnight trypsinization at 37°C, the supernatant was collected and vacuum dried. The digests were diluted to a final peptide concentration of 200 ng/µL in 0.1% trifluoracetic acid.

Samples were analyzed by nano-LC (Ultimate 3000RS, Thermo Fisher Scientific, Germering, Germany). After preconcentration and washing of the samples on a C18 trap column (1 mm ×300 µm i.d., Thermo Fisher Scientific), they were loaded onto a C18 column (PepMap C18, 75 mm ID × 250 mm, 2 µm particle and 100 Å pore size, Thermo Fisher Scientific) using a linear 90 min gradient (4%–38% ACN/H20; 0.1% formic acid) at a flow rate of 250 nL/min. The separation of the peptides was monitored by a UV detector (absorption at 214 nm). The nano-LC was coupled to a nanospray source of a Q Exactive HF mass spectrometer (Thermo Fisher Scientific, Bremen, Germany), which was operated in the data-dependent acquisition (DDA) mode. Full scan MS spectra (*m*/*z* 375–1,500) in profile mode were acquired in the Orbitrap with a resolution of 60,000 after the accumulation of an Automatic Gain Control (AGC) target of 3 × 106. The top 20 peptide signals (charge state 2+ and higher) were isolated (1.4 *m/z* window) and fragmented by higher-energy collision (normalized collision energy, 28.0) and measured in the Orbitrap with an AGC target of 50,000 and a resolution of 15,000. Maximum fill times were 60 ms for the full scans and 50 ms for the MS/MS scans. The dynamic exclusion was activated; after the first time a precursor was selected for fragmentation, it was excluded for a period of 40 seconds using a relative mass window of 10 ppm. Lock mass correction was activated to improve the mass accuracy of the survey scan. After each measurement, the column was rinsed with a blank to minimize carryover.

### DDA data processing and analysis

DDA data were analyzed using MaxQuant 1.6.1.0 (Max Planck Institute for Chemistry, Mainz, Germany) with default settings unless indicated otherwise. A maximum of two missed cleavages was allowed. Both methionine oxidation and acetylation of the protein n-termini were set as variable modification, carbamidomethylation was set as a fixed modification of cysteine, and trypsin was set as the enzyme. The *Klebsiella pneumoniae* refseq protein database was used, downloaded on 13 April 2021 (43). Due to the large number of sequenced genomes available at the time, this database provided a broad representation of protein variants. Key proteins not detected in our MS data (e.g., OmpK35) were manually confirmed to be present in the database, ensuring completeness of the annotation. The label-free quantification option with matching between runs was used in MaxQuant. The quantitative values for all identified protein groups were further analyzed in Perseus 1.6.1.2 (Max Planck Institute for Chemistry, Mainz, Germany). The data were annotated and log2 transformed. Missing values were replaced by values from a down-shifted normal distribution of the intensities (“imputed”). The data were subsequently checked for the detection of β-lactamase proteins for which the genes were identified by whole-genome sequencing, as well as for porins OmpK35, OmpK36, OmpK26, OmpW, and OmpX, outer membrane protein OmpK34, and the efflux systems TolC, AcrAB, and OqxAB involved in β-lactam resistance. Plasmid copy numbers from the Unicycler assembly files and log2-transformed protein quantification values were visualized as heatmaps using GraphPad Prism (GraphPad Software, San Diego, USA).

### Meropenem resistance selection

From overnight incubated blood agar plates, colonies of OXA-48-positive (*n* = 1) and OXA-48-negative (*n* = 3) isolates with low (0.25–2 µg/mL) or intermediate (4–8 µg/mL) MICs for meropenem were suspended in 5 mL Brain Heart Infusion (BHI) broth. From this overnight culture, 500 µL was used to inoculate 5 mL of BHI broth containing concentrations of meropenem at 0.25× MIC, 0.5× MIC, 1× MIC, 2× MIC, and 4× MIC of each particular isolate. After overnight growth, the liquid culture containing the highest meropenem concentration showing visible growth was used to inoculate another culture selection round. For all subsequent rounds, meropenem concentrations of 0.5×, 1×, 2×, and 4× the meropenem concentration of the previous round were used, with a maximum meropenem concentration of 32 µg/mL. Meropenem selection was stopped once a liquid culture had reached visible growth at a meropenem concentration of 32 µg/mL or after a total of 11 rounds of exposure.

For each isolate, the final generation was serially diluted from undiluted to 10^−6^ and plated on MHB agar plates containing meropenem at 0.5×, 1×, 2×, and 4× the concentration of the selected stock. For each meropenem-exposed isolate, three colonies growing on the plate containing the highest concentration of meropenem and the highest dilution of bacterial suspension were selected and stored in a glycerol stock at −80°C until further use. For each strain, one out of three colonies was randomly selected and analyzed by imipenem and meropenem BMD, Illumina and Nanopore sequencing, and LC-MS/MS according to the above-mentioned protocols. The meropenem-exposed isolates are depicted as the isolate number followed by the rounds of exposure to the meropenem resistance selection.

## Data Availability

Sequence data of the 15 clinically obtained *K. pneumoniae* isolates and 4 meropenem-exposed isolates are available under BioProject ID PRJNA 1241873 and BioSample accession numbers SAMN47572891 through SAMN47572909 in the NCBI BioProject database. The mass spectrometry proteomics data have been deposited in the ProteomeXchange Consortium via the PRIDE (44) partner repository with the dataset identifier PXD061983. All other data are included in this article or available as Supplemental material.

## SUPPLEMENTAL MATERIAL

Supplemental material aac.00107-25-s0001.docx Tables S1 to S3; Fig. S1 and S2.

ASM does not own the copyrights to Supplemental Material that may be linked to, or accessed through, an article. The authors have granted ASM a non-exclusive, world-wide license to publish the Supplemental Material files. Please contact the corresponding author directly for reuse.
