# Supplementary material for: Proteogenomic analysis demonstrates increased blaOXA-48 copy numbers and OmpK36 loss as contributors to carbapenem resistance in Klebsiella pneumoniae
Source: Antimicrob Agents Chemother. 2025 Jun 13;69(7):e00107-25. doi: 10.1128/aac.00107-25 (PMC12217476; doi:10.1128/aac.00107-25)
Supplement: Supplemental material — Tables S1 to S3; Fig. S1 and S2. [file aac.00107-25-s0001.docx]

**Table S1. Characterization of all 74 *Klebsiella pneumoniae* isolates.**

| **Isolate ID** | **MLVA-type** | **IMI** | **MEM** | ***bla*_OXA-48-like_** | ***bla*_NDM_** |
| --- | --- | --- | --- | --- | --- |
| **1** | A | 2 | 8 | + | - |
| **2** | A | 4 | 16 | + | - |
| **3** | A | 16 | 16 | + | - |
| **4** | A | 2 | 16 | + | - |
| **5** | A | 16 | 16 | + | - |
| **6** | A | 16 | 16 | + | - |
| **7** | A | 128 | >32 | + | - |
| **8** | A | 128 | >32 | + | - |
| **9** | A | 4 | 16 | + | - |
| **10** | A | 4 | 32 | + | - |
| **11** | A | 4 | 16 | + | - |
| **12** | A | 0,25 | 0,25 | - | - |
| **13** | A | <0.125 | 1 | - | - |
| **14** | A | 0,25 | 0,5 | - | - |
| **15** | B | 128 | >32 | + | + |
| **16** | B | 0,5 | 2 | - | - |
| **17** | B | 0,5 | 1 | - | - |
| **18** | B | 1 | 1 | - | - |
| **19** | B | 0,5 | 2 | - | - |
| **20** | C | 128 | >32 | + | - |
| **21** | C | 128 | >32 | + | - |
| **22** | C | 2 | 8 | - | - |
| **23** | C | 2 | 8 | - | - |
| **24** | C | 2 | 8 | + | - |
| **25** | D | 4 | 8 | - | - |
| **26** | E | <0.125 | 1 | - | - |
| **27** | E | 0,25 | 1 | - | - |
| **28** | F | 8 | 4 | + | - |
| **29** | F | 2 | 4 | + | - |
| **30** | unique | 64 | >32 | + | + |
| **31** | A | 2 | 16 | + | - |
| **32** | A | 4 | 16 | + | - |
| **33** | A | 4 | 16 | + | - |
| **34** | A | 8 | 16 | + | - |
| **35** | A | 4 | 32 | + | - |
| **36** | A | 4 | 16 | + | - |
| **37** | A | 4 | 16 | + | - |
| **38** | A | 4 | 32 | + | - |
| **39** | A | 2 | 16 | + | - |
| **40** | A | 8 | 32 | + | - |
| **41** | A | 2 | 16 | + | - |
| **42** | A | 4 | 16 | + | - |
| **43** | A | 4 | 16 | + | - |
| **44** | A | 2 | 8 | + | - |
| **45** | A | 2 | 16 | + | - |
| **46** | A | 4 | 16 | + | - |
| **47** | A | 2 | 16 | + | - |
| **48** | A | 4 | 16 | + | - |
| **49** | A | 4 | 16 | + | - |
| **50** | A | 2 | 16 | + | - |
| **51** | A | 2 | 16 | + | - |
| **52** | A | 2 | 16 | + | - |
| **53** | A | 2 | 16 | + | - |
| **54** | A | 4 | 16 | + | - |
| **55** | A | 4 | 16 | + | - |
| **56** | A | 4 | 16 | + | - |
| **57** | A | 4 | 16 | + | - |
| **58** | A | 4 | 16 | + | - |
| **59** | A | 2 | 16 | + | - |
| **60** | A | 2 | 32 | + | - |
| **61** | A | 64 | >32 | + | - |
| **62** | A | 128 | >32 | + | - |
| **63** | A | 4 | 16 | + | - |
| **64** | A | 4 | 16 | + | - |
| **65** | A | 4 | 32 | + | - |
| **66** | A | 8 | 16 | + | - |
| **67** | A | 4 | 32 | + | - |
| **68** | A | 4 | 16 | + | - |
| **69** | A | 32 | 32 | + | - |
| **70** | A | 2 | 16 | + | - |
| **71** | C | 64 | >32 | + | - |
| **72** | A | 8 | 1 | - | - |
| **73** | B | 0,25 | 1 | - | - |
| **74** | B | 0,25 | 1 | - | - |

IMI = imipenem MIC (µg/ml), MEM = meropenem MIC (µg/ml)

**Table S2. Genomic characterization of all 15 sequenced *K. pneumoniae* isolates.**

| **ID** | **MLVA** | **MLST** | **IMI** | **MEM** | **OXA-48** | **NDM** | **Resistance genes on chromosome** | **Resistance genes on plasmid** | **Plasmid size (bp)** | **Plasmid copy number Unicycler** |
| --- | --- | --- | --- | --- | --- | --- | --- | --- | --- | --- |
| **26** | E | ST-525 | <0.125 | 1 | - | - | **blaCTX-M-15**, **blaSHV-11**, oqxA, oqxB | IncFII: aac(3)-IIa, aac(6')-Ib-cr, **blaCTX-M-15**, **blaOXA-1**, **blaTEM-1B**, catB4, tet(A) | 91,539 | 1.62 |
| **12** | A | ST-101 | 0.25 | 0.25 | - | - | 3x **blaCTX-M-15, blaSHV-1**, oqxA, oqxB | no Inc: aac(3)-IIa, aac(6')-Ib, aac(6')-Ib-cr, aadA1, **blaOXA-9**, **blaTEM-1A** | 94,971 | 1.05 |
| **73** | B | ST-15 | 0.25 | 1 | - | - | **blaSHV-28**, catA1, fosA6, oqxA | IncFIB(Mar)/IncHI1B: aac(3)-Ila, ant(3'')-Ia, **blaTEM-1B**, dfrA1, sul1 | 355,719 | 1,01 |
|  |  |  |  |  |  |  |  | IncFII: aac(6')-Ib-cr, **blaCTX-M-15**, **blaOXA-1**, **blaTEM-1B**, catB4, tet(A) | 88,881 | 2.59 |
| **17** | B | ST-15 | 0.5 | 1 | - | - | **blaSHV-28**, catA1, fosA6, oqxA, oqxB | IncFIB(Mar)/IncHI1B: aac(3)-IIa, ant(3'')-Ia, **blaTEM-1B**, dfrA1, sul1 | 355,719 | 0.77 |
|  |  |  |  |  |  |  |  | IncFII: aac(6')-Ib-cr, **blaCTX-M-15**, **blaOXA-1**, **blaTEM-1B**, catB4, tet(A) | 88,881 | 2.74 |
| **29** | F | ST-395 | 2 | 4 | + | - | **blaSHV-11**, fosA, oqxA, oqxB | IncR: aac(6')-Ib-cr, **blaOXA-1**, catB4, dfrA1, sul1, tet(A) | 41,918 | 1.38 |
|  |  |  |  |  |  |  |  | IncL(pOXA-48): **blaOXA-48** | 48,734 | 1.80 |
| **24** | C | ST-147 | 2 | 8 | + | - | **blaSHV-11**, fosA, oqxA, oqxB | IncHI1B: ARR-3, aac(3)-IId, aph(3'')-Ib, aph(6)-Id, **blaCTX-M-15**, **blaTEM-1B**, 2x catA1, dfrA5, ere(A), ere(B), sul1, sul2 | 198,404 | 1.08 |
|  |  |  |  |  |  |  |  | IncFIB(pQil): aac(6')-Ib, aac(6')-Ib-cr, aadA1, **blaOXA-9** | 92,594 | 1.12 |
|  |  |  |  |  |  |  |  | IncL(pOXA-48): **blaOXA-48** | 63,589 | **0.98** |
|  |  |  |  |  |  |  |  | No Inc: **blaCTX-M-15**, **blaTEM-1B** | 23,367 | 1.07 |
| **59** | A | ST-101 | 2 | 16 | **+** | - | 3x b**laCTX-M-15, blaSHV-1**, oqxA, oqxB | IncFIB(pQil): aac(6')-Ib, aac(6')-Ib-cr, aadA1, **blaOXA-9** | 92,696 | 1.15 |
|  |  |  |  |  |  |  |  | IncFIA(HI1)/IncR: aac(3)-IIa, aac(6')-Ib, aac(6')-Ib-cr, aadA1, **blaOXA-9** | 38,702 | 1.03 |
|  |  |  |  |  |  |  |  | IncL(pOXA-48): **blaOXA-48** | 63,589 | **0.77** |
| **9** | A | ST-101 | 4 | 16 | **+** | - | 3x **blaCTX-M-15, blaSHV-1**, oqxA, oqxB | IncFIB(pQil): aac(6')-Ib, aac(6')-Ib-cr, aadA1, **blaOXA-9** | 92,696 | 1.31 |
|  |  |  |  |  |  |  |  | IncFIA(HI1)/IncR: aac(3)-IIa, aac(6')-Ib, aac(6')-Ib-cr, aadA1, **blaOXA-9** | 38,702 | 1.20 |
|  |  |  |  |  |  |  |  | IncL(pOXA-48): **blaOXA-48** | 64,366 | **1.03** |
|  |  |  |  |  |  |  |  | no Inc: tet(D) | 3,747 | 0.87 |
| **35** | A | ST-101 | 4 | 32 | **+** | - | 3x **blaCTX-M-15, blaSHV-**1, oqxA, oqxB | IncFIA(HI1)/IncR: aac(6')-Ib, aac(6')-Ib-cr, aadA1, **blaOXA-9**, **blaSHV-1**, tet(D) | 61,176 | 0.99 |
|  |  |  |  |  |  |  |  | IncL(pOXA-48): **blaOXA-48** | 63,589 | **0.8** |
| **72** | A | ST-101 | 8 | 1 | - | - | 2x **blaCTX-M-15**, **blaSHV-1**, oqxA, oqxB | IncR: **blaCTX-M-15**, tet(D) | 38,546 | 1.33 |
| **28** | F | ST-395 | 8 | 4 | + | - | **blaSHV-11**, fosA, oqxA, oqxB | IncR: aac(3)-IIa, aac(6')-Ib-cr, **blaCTX-M-15**, **blaOXA-1**, **blaTEM-1B**, catB4, dfrA1, qnrS1, sul1 | 61,928 | 5.23 |
|  |  |  |  |  |  |  |  | IncL(pOXA-48): **blaOXA-48** | 63,589 | **5.27** |
| **3** | A | ST-101 | 16 | 16 | **+** | - | 3x **blaCTX-M-15, blaSHV-1**, oqxA, oqxB | IncFIB(pQil): aac(6')-Ib, aac(6')-Ib-cr, aadA1, **blaOXA-9** | 92,696 | 1.67 |
|  |  |  |  |  |  |  |  | IncFIA(HI1)/IncR: aac(3)-IIa, aac(6')-Ib, aac(6')-Ib-cr, aadA1, **blaOXA-9**, **blaSHV-1**, tet(D) | 64,592 | 1.55 |
|  |  |  |  |  |  |  |  | IncL(pOXA-48): **blaOXA-48** | 63,589 | **1.57** |
| **7** | A | ST-101 | 128 | 128 | **+** | - | 2x **blaCTX-M-15, blaSHV-1,** oqxA, oqxB | no Inc:aac(3)-lla, **blaTEM-1A** | 91,924 | 4.42 |
|  |  |  |  |  |  |  |  | IncL(pOXA-48): **blaOXA-48** | 63,252 | **5.97** |
| **62** | A | ST-101 | 128 | 128 | **+** | - | 2x **blaCTX-M-15**, **blaSHV-1**, oqxA, oqxB | IncFIA(HI1)/IncR: aac(3)-IIa, aac(6')-Ib, aac(6')-Ib-cr, aadA1, **blaOXA-9**, **blaSHV-1**, tet(D) | 65,424 | 4.55 |
|  |  |  |  |  |  |  |  | IncL(pOXA-48): **blaOXA-48** | 63,589 | **6.66** |
|  |  |  |  |  |  |  |  | no Inc: **blaCTX-M-15** | 10,405 | **8.66** |
| **15** | B | ST-15 | 128 | 128 | + | + | **blaSHV-28**, fosA6, oqxA, oqxB | IncFII(K): aac(3)-IIa, **blaCTX-M-15**, dfrA30, tet(A) | 139,040 | 1.19 |
|  |  |  |  |  |  |  |  | IncFII(Yp): **blaNDM-1**, rmtC, sul1 | 110,777 | **1.60** |
|  |  |  |  |  |  |  |  | IncL(pOXA-48): **blaOXA-48** | 63,499 | **0.73** |

IMI = imipenem MIC (µg/ml), MEM = meropenem MIC (µg/ml), β-lactamase genes are depicted in bold and *bla*_OXA-48_ positive isolates are underlined.

**Table S3. Genomic characterization of native and meropenem exposed isolates.**

| **ID** | **IMI** | **MEM** | **OXA-48** | **Resistance genes on chromosome** | **Resistance genes on plasmid** | **Plasmid size (bp)** | **Plasmid copy number** |
| --- | --- | --- | --- | --- | --- | --- | --- |
| 73 | 0.25 | 1 | - | **blaSHV-28**, catA1, fosA6, oqxA | IncFIB(Mar)/IncHI1B: aac(3)-Ila, ant(3'')-Ia, **blaTEM-1B**, dfrA1, sul1 | 355,719 | 1,01 |
|  |  |  |  |  | IncFII: aac(6')-Ib-cr, **blaCTX-M-15**, **blaOXA-1**, **blaTEM-1B**, catB4, tet(A) | 88,881 | 2.59 |
| 73.9 | 8 | 32 | - | **blaSHV-28**, catA1, fosA6, oqxA | IncFIB(Mar)/IncHI1B: aac(3)-Ila, ant(3'')-Ia, **blaTEM-1B**, dfrA1, sul1 | 355,735 | 0.71 |
|  |  |  |  |  | IncFII: aac(6')-Ib-cr, **blaCTX-M-15**, **blaOXA-1**, **blaTEM-1B**, catB4 | 83,409 | 17.23 |
|  |  |  |  |  | No Inc: tet(A) | 5,472 | 10.75 |
| 17 | 0.5 | 1 | - | **blaSHV-28**, catA1, fosA6, oqxA, oqxB | IncFIB(Mar)/IncHI1B: aac(3)-IIa, ant(3'')-Ia, **blaTEM-1B**, dfrA1, sul1 | 355,719 | 0.77 |
|  |  |  |  |  | IncFII: aac(6')-Ib-cr, **blaCTX-M-15,** **blaOXA-1**, **blaTEM-1B**, catB4, tet(A) | 88,881 | 2.74 |
| 17.11 | 8 | 32 | - | **blaSHV-28**, catA1, fosA6, oqxA, oqxB | IncFIB(Mar): dfrA1 | 137,117 | 0.37 |
|  |  |  |  |  | IncHI1B: aac(3)-IIa | 43,084 | 0.40 |
|  |  |  |  |  | No Inc: ant(3'')-Ia, sul1 | 18,798 | 0.41 |
|  |  |  |  |  | No Inc: **blaTEM-1B** | ,779 | 0.4 |
|  |  |  |  |  | IncFII: aac(6')-Ib-cr, **blaCTX-M-15,** **blaOXA-1**, **blaTEM-1B**, catB4, tet(A) | 88,881 | 9.35 |
| 24 | 2 | 8 | + | **blaSHV-11**, fosA, oqxA, oqxB | IncHI1B: ARR-3, aac(3)-IId, aph(3'')-Ib, aph(6)-Id, **blaCTX-M-15**, **blaTEM-1B**, 2x catA1, dfrA5, ere(A), ere(B), sul1, sul2 | 198,404 | 1.08 |
|  |  |  |  |  | IncFIB(pQil): aac(6')-Ib, aac(6')-Ib-cr, aadA1, **blaOXA-9** | 92,594 | 1.12 |
|  |  |  |  |  | **IncL(pOXA-48): blaOXA-48** | 63,589 | **0.98** |
|  |  |  |  |  | No Inc: **blaCTX-M-15**, **blaTEM-1B** | 23,367 | 1.07 |
| 24.4 | >128 | 128 | + | **blaSHV-11**, fosA, oqxA, oqxB | IncHI1B: ARR-3, aac(3)-IId, aph(3'')-Ib, aph(6)-Id, **blaCTX-M-15**, **blaTEM-1B**, 2x catA1, dfrA5, ere(A), ere(B), sul1, sul2 | 198,404 | 1.75 |
|  |  |  |  |  | IncFIB(pQil): aac(6')-Ib, aac(6')-Ib-cr, aadA1, **blaOXA-9** | 92,594 | 2.30 |
|  |  |  |  |  | **IncL(pOXA-48): blaOXA-48** | 63,589 | **3.72** |
|  |  |  |  |  | No Inc: **blaCTX-M-15**, **blaTEM-1B** | 23,367 | 3.09 |
| 72 | 8 | 1 | - | 2x **blaCTX-M-15**, **blaSHV-1**, oqxA, oqxB | IncR: **blaCTX-M-15**, tet(D) | 38,546 | 1.33 |
| 72.11 | 8 | 32 | - | 2x **blaCTX-M-15**, **blaSHV-1**, oqxA, oqxB | IncR: **blaCTX-M-15**, tet(D) | 38,630 | 7.06 |

IMI = imipenem MIC (µg/ml), MEM = meropenem MIC (µg/ml), β-lactamase genes are depicted in bold and *bla*_OXA-48_ positive isolates are underlined.


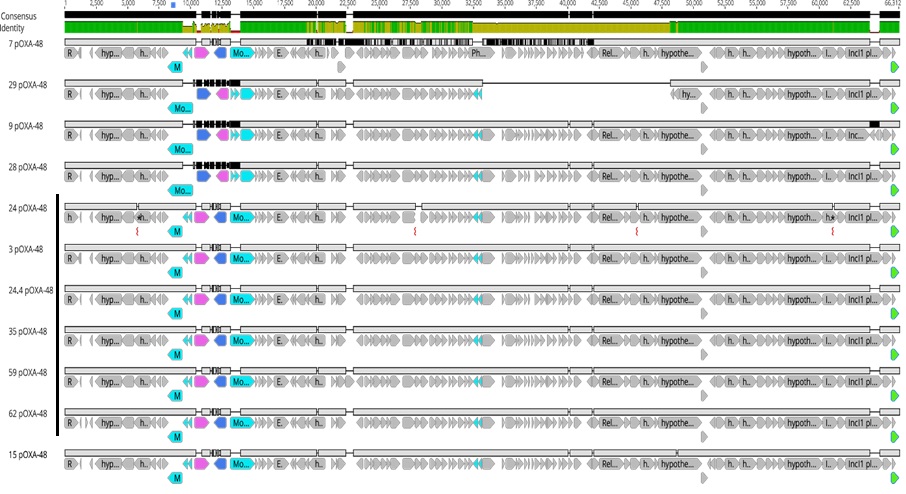


**Figure S1.** Visualization of the IncL(pOXA-48) plasmid of all 10 clinically obtained *bla*_OXA-48_ positive isolates and one meropenem exposed *bla*_OXA-48_ positive isolate. The black vertical lines shows the 100% identical pOXA-48 sequences of isolates 3, 24, 35, 59, 62 and meropenem exposed isolate 24.4. The *bla*_OXA48_ gene is indicated in violet, a transcriptional regulator in blue, mobile elements in light blue and the replicon IncL(pOXA-48) in green.


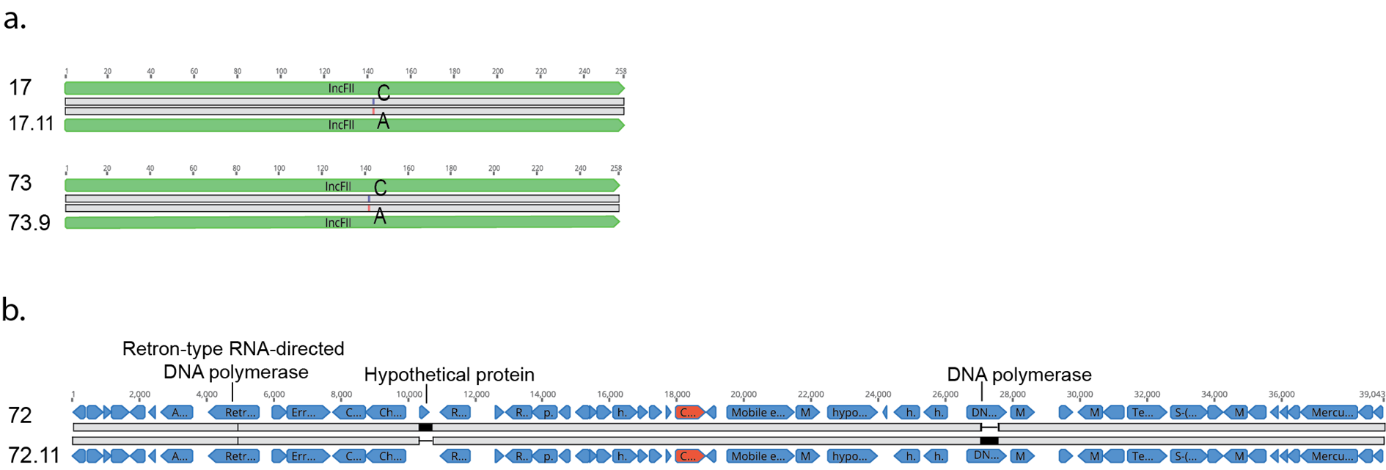


**Figure S2.** Alterations in IncFII and IncR plasmids after exposure to meropenem. (a) IncFII operon before (isolate 17 and 73) and after (isolate 17.11 and 73.9) exposure to meropenem. (b) A representation of the IncR plasmid of isolate 72 and 72.11 with gene products that were altered after exposure to meropenem indicated. *bla*_CTX-M-15_ is indicated in red.
